# Relationship between gut microbiota and vascular calcification in hemodialysis patients

**DOI:** 10.1080/0886022X.2022.2148538

**Published:** 2023-01-12

**Authors:** Wen-Han Bao, Wen-Ling Yang, Chun-Yan Su, Xin-Hong Lu, Lian He, Ai-Hua Zhang

**Affiliations:** a Department of Nephrology, Peking University Third Hospital, Beijing, PR China; b Department of Nephrology, Xuanwu Hospital Capital Medical University, Beijing, PR China

**Keywords:** End-stage renal disease, hemodialysis, vascular calcification, gut microbiota

## Abstract

**Introduction:**

Vascular calcification (VC) is an independent risk factor for cardiovascular mortality in end-stage renal disease (ESRD) patients. The pathogenesis of VC is complicated and unclear. Uremic toxins produced by gut microbiota can promote VC. This study aims to identify the differences in gut microbiota between the different VC groups and the main bacteria associated with VC in hemodialysis (HD) patients in an attempt to open up new preventive and therapeutic approaches and define the probable mechanism for VC in HD patients in the future.

**Methods:**

A total of 73 maintenance HD patients were enrolled in this cross-sectional study. According to the abdominal aortic calcification (AAC) scores, the participants were divided into the high AAC score group and the low AAC score group. High-throughput sequencing of the gut microbiota was performed and the results were evaluated by alpha diversity, beta diversity, species correlation, and model predictive analyses.

**Results:**

The prevalence of VC was 54.79% (40/73) in the study. The majority of phyla in the two groups were the same, including *Firmicutes*, *Actinobacteriota*, *Proteobacteria*, and *Bacteroidota*. The microbial diversity in the high AAC score group had a decreasing trend (*p* = 0.050), and the species abundance was significantly lower (*p* = 0.044) than that in the low AAC score group. The HD patients with high AAC scores showed an increased abundance of *Proteobacteria* and decreased abundances of *Bacteroidota* and *Synergistota* at the phylum level; increased abundances of *Escherichia-Shigella, Ruminococcus_gnavus_group,* and *Lactobacillus;* and decreased abundances of *Ruminococcus* and *Lachnospiraceae_NK4A136_group* at the genus level (*p<*0.05). *Escherichia-Shigella* and *Ruminococcus_gnavus_group* were positively correlated with VC, and *Ruminococcus, Adlercreutzia*, *Alistipes,* and *norank_f__Ruminococcaceae* were negatively correlated with VC. *Escherichia-Shigella* had the greatest influence on VC in HD patients, followed by *Ruminococcus* and *Butyricimonas.*

**Conclusions:**

Our results provide clinical evidence that there was a difference in gut microbiota between the different VC groups in HD patients. *Escherichia–Shigella,* a lipopolysaccharide (LPS)-producing bacterium, was positively correlated with VC and had the greatest influence on VC. *Ruminococcus,* a short-chain fatty acid (SCFA)-producing bacterium, was negatively correlated with VC and had the second strongest influence on VC in HD patients. The underlying mechanism is worth studying. These findings hint at a new therapeutic target.

## 1. Introduction

Chronic kidney disease (CKD) is a global public health problem [1]. The prevalence of CKD in Chinese adults is over 10% [2]. The number of patients progressing to end-stage renal disease (ESRD) is increasing year by year. Cardiovascular disease (CVD) is the leading cause of death among patients with ESRD [3,4]. Vascular calcification (VC) is an independent risk factor for cardiovascular mortality and all-cause mortality in ESRD patients. However, the pathogenesis of VC is complicated and unclear [5].

The gut microbiota constitutes a complex and large microecosystem in the human digestive tract [6]. CKD can cause an imbalance in gut microbiota and impaired intestinal barrier function, resulting in gut microbiota displacement, excessive uremic toxin production, and a systemic inflammatory response, thus accelerating the progression of CKD [1]. Uremic toxins, such as indoxyl sulfate (IS), p-cresyl sulfate (PCS), and trimethylamine N-oxide (TMAO), can promote VC [7–9]. Luo et al. found that gut microbiota disturbance is associated with prognosis in ESRD patients [10]. Recently, researchers have expected to reduce VC and improve cardiovascular prognosis by altering gut microbiota and reducing associated uremic toxins in ESRD patients, but the results of intervention studies have been inconsistent [1,11]. It is not clear which bacteria or uremic toxins have the greatest impact on VC and survival in ESRD patients.

In addition, it was reported that the diversity and taxa of gut microbiota were different between nondialysis CKD and dialysis patients or between hemodialysis (HD) and peritoneal dialysis (PD) patients [12,13]. These differences may be associated with systemic inflammation and the mode of renal replacement therapy [12,13]. A study in Chinese ESRD patients found that the prevalence of VC in HD patients was higher than that in PD patients [14].

Thus, in this study, we expect to find the bacteria that have the greatest influence on VC in HD patients to provide precise intervention and improve the prognosis of patients. Meanwhile, we also expected to identify the metabolic pathways related to the corresponding microbiota to explore the mechanisms of VC. Currently, similar clinical studies are rare.

## 2. Materials and methods

### 2.1. Participants

**Figure 1.**
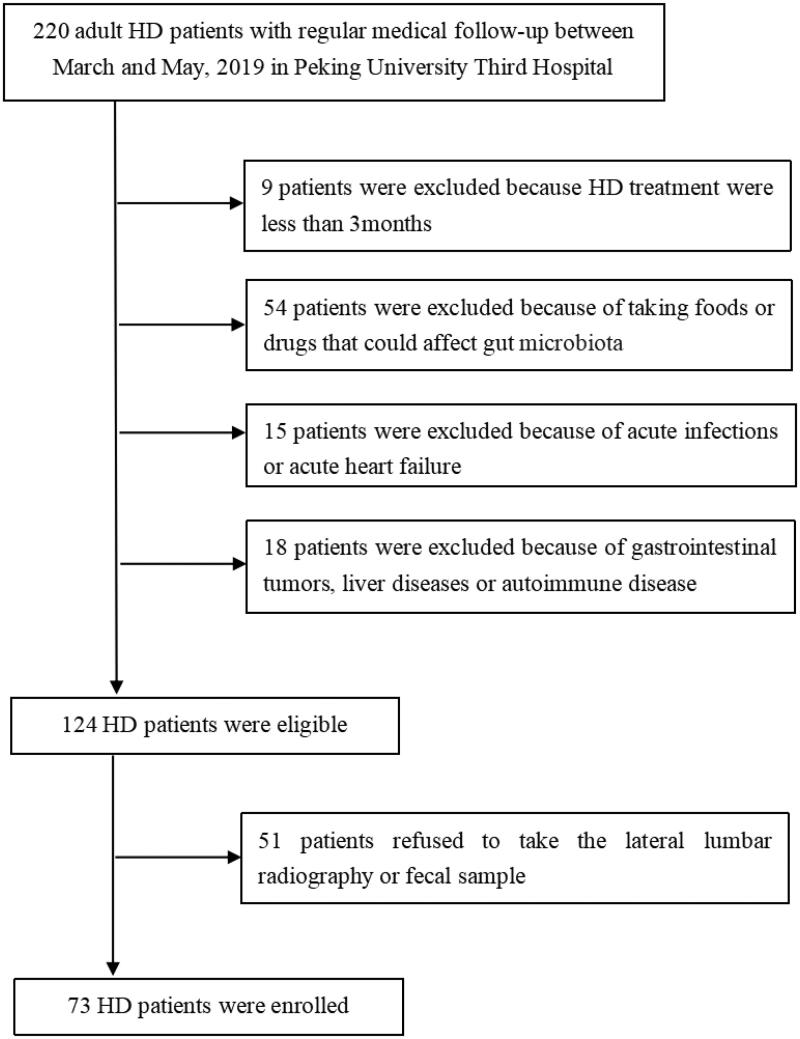
Flowchart of the study. HD: hemodialysis patients.

This cross-sectional study screened 220 maintenance HD patients and enrolled 73 patients in the Peking University Third Hospital between March and May 2019. Patients were eligible for inclusion if they (1) were aged >18 years old and (2) had been on HD for at least 3 months, with no changes in the dialysis mode and volume in the past 1 month. The exclusion criteria included (1) acute-phase infections of the respiratory tract, digestive tract, or urinary tract; (2) intake of foods and drugs (e.g. prebiotics, probiotics, and synbiotics) that are involved in the regulation of the gut microbiota in the past 6 months; (3) administration of antibiotics, hormones, or immunosuppressive agents in the past 3 months; (4) acute heart failure; (5) gastrointestinal tumors or a history of abdominal organ surgery; (6) liver disease or abnormal liver function; (7) autoimmune disease; and (8) refusal to participate in the study. All individuals who participated in this study provided written informed consent, and the protocol was approved by the Peking University Third Hospital Medical Science Research Ethics Committee (approval no. IRB00006761 – M2019003). A flowchart of patient recruitment for the study is shown in Figure 1.

### 2.2. Clinical and biochemical data collection

Data collected included patient demographics, such as age, sex, body mass index (BMI), history of diabetes mellitus (DM), history of CVD, history of hypertension, history of smoking, HD vintage, and medication history, such as H2 receptor blockers, proton pump inhibitors (PPI), phosphate binders, and oral iron supplementation. Laboratory data were collected at baseline from patients after fasting for 12 h, including hemoglobin, creatinine, albumin, corrected calcium, phosphorus, intact parathyroid hormone (iPTH), alkaline phosphatase (ALP), ultrasensitive C reactive protein (usCRP), and lipid profile. Patients were also required to give 3-d records of diets on the basis of usual dietary habits in the week of fecal sample collection. The food records were converted into the amounts of carbohydrates, proteins, fats, and fibers.

### 2.3. Fecal sample collection, DNA extraction, and 16S rRNA sequence analysis

#### 2.3.1. Fecal sample collection and DNA extraction

Fecal samples collected from the HD patients were immediately frozen in liquid nitrogen and stored at −80 °C until use. Stool samples were transported to the Shanghai Majorbio Bio-Pharm Technology Co., Ltd., Laboratory on dry ice within three months following freezing. Microbial DNA was extracted using an OMEGA-soil DNA Kit (Omega Bio-Tek, Norcross, GA). The V1–V3 regions of the *16S rRNA* gene were amplified by PCR (95 °C for 3 min; 27 cycles at 95 °C for 30 s, 55 °C for 30 s, and 72 °C for 45s; and a final extension at 72 °C for 10 min) with primers (338 forward primer: 5′-ACTCCTACGGGAGGCAGCAG-3′; 806 reverse primer: 5′-GGACTACHVGGGTWTCTAAT-3′). All samples were amplified in triplicate. The PCR products were extracted from 2% agarose gels, purified using an AxyPrep DNA Gel Extraction Kit (Axygen Biosciences, Union City, CA) according to the manufacturer’s instructions and quantified using a Quantus^™^ Fluorometer (Promega, Madison, WI).

#### 2.3.2. Illumina MiSeq sequencing

Purified amplicons were pooled in equimolar amounts and paired-end sequenced on an Illumina MiSeq PE300 platform (Illumina, San Diego, CA) according to the standard protocols by Majorbio Bio-Pharm Technology Co., Ltd. (Shanghai, China).

#### 2.3.3. 16S rRNA sequence analysis

Fastp software was used for quality control of the original sequence and Flash software was used for stitching. Usearch software version 7.0 (http://drive5.com/uparse/) was used for operational taxonomic unit (OTU) clustering of sequences based on 97% similarity and chimera elimination. The RDP classifier (http://rdp.cme.msu.edu/) was used to compare each sequence with the sequences included in the Silva database (SSU138). The threshold was set to 70%, and the results of species classification annotations were obtained. The sample species composition was analyzed based on the annotation results.

### 2.4. Assessment of vascular calcification (VC)

VC was imaged within 1 week of enrollment: abdominal aortic calcification (AAC) by lateral lumbar radiography with Kauppila scoring [15]. Lateral lumbar radiographs were taken by X-ray radiography (GE 646 HD). For evaluation of AAC, we used a semiquantitative scoring system, as described by Kauppila et al. Briefly, the abdominal aorta adjacent to the first four lumbar vertebrae was divided into four segments using the midpoint of each intervertebral space as a boundary. Anterior and posterior aortic wall segments were evaluated separately. Calcific deposits were graded on a scale of 0–3 at each segment as follows: 0 = no calcific deposits, 1 = small, scattered calcific deposits filling less than one-third of the aortic wall, 2 = one-third to two-thirds of the aortic wall calcified, and 3 = at least two-thirds of the aortic wall calcified. The grades of the eight aortic segments were summed in the Kauppila calcification score (the antero-posterior severity score), ranging from 0 to 24 points. Two independent observers scored all lateral lumbar X-rays. Both observers were blinded to the clinical and laboratory patient data. The two scores were averaged as the AAC for the patient. If the score difference was more than 5 points, the x-ray was scored by the third observer, and the average of the three scores was taken. Patients were allocated to the high AAC score group if they had an AAC score of ≥4 and the low AAC score group if they had an AAC score of <4 [16].

### 2.5. Bioinformatics and statistical analysis

The alpha diversity analysis was calculated using the Chao1, Shannon, and coverage indices based on the OTU profiles to reflect species diversity. The Wilcoxon rank-sum test was used to analyze the differences in alpha diversity and abundance between the two groups. A *p* value of <0.05 was considered significant. Beta diversity analysis or between-group differences in species composition were analyzed by principal coordinates analysis (PCoA) based on Bray–Curtis distance. Statistical significance was determined using permutational multivariate analysis of variance (PERMANOVA). Correlation analysis of species was carried out by correlation heatmaps. The model predictive analysis was carried out by random forest analysis. Phylogenetic investigation of communities by reconstruction of unobserved states (PICRUSt) was used to perform Kyoto Encyclopedia of Genes and Genomes (KEGG) pathway and abundance analysis based on different numbers of 16S rRNA copies.

The results are expressed as proportions (percentages) for categorical variables, means ± standard deviations for continuous normally distributed variables, and medians with interquartile ranges for continuous non-normally distributed variables. Student’s t test was used to compare differences between the two groups for normally distributed data, while the Mann–Whitney U test was used for non-normal data. Categorical data were compared using the chi-square test. Binary logistic analysis was performed to determine the independent influencing factors of VC (using backward conditional). All analyses were two-tailed and *p* < 0.05 was considered statistically significant. SPSS Software version 25.0 (SPSS Inc., Chicago, IL), was used for all statistical analyses.

## 3. Results

### 3.1. Basic information

Seventy-three HD patients were recruited according to the inclusion and exclusion criteria. The median AAC scores in the high AAC score group and in the low AAC score group were 8.00 (6.00, 11.75) and 1.00 (0.00, 2.00), respectively. The demographic and clinical characteristics of the patients in the high and low AAC score groups are shown in Table 1. The median age of the patients in the high AAC score group was 67.00 (60.00, 74.00) years. The median HD vintage was 82.85 (52.85, 127.82) months. There were significant differences in age, HD vintage, and serum creatinine. There were no significant differences in sex, BMI, history of DM, history of CVD, history of hypertension, history of smoking, medication history, hemoglobin, serum calcium, serum phosphate, serum albumin, serum iPTH, serum usCRP, serum ALP, serum lipid profile, or the amounts of carbohydrates, proteins, fats, and fibers between the high and low AAC score groups in HD patients (see Table 1).

**Table 1.** Comparison of basic parameters between the high and low AAC score groups in hemodialysis patients.

Items	The high AAC score group（*n* = 40）	The low AAC score group（*n* = 33）	*p* Value
Age (years)	67.00 (60.00, 74.00)	55.00 (43.00, 67.00)	0.001
Male/female (*n*)	24/16	23/10	0.389
BMI (kg/m^2^)	21.96 (20.42, 25.39)	22.90 (21.06, 25.14)	0.325
DM (yes/no)	16/24	11/22	0.557
CVD (yes/no)	12/28	4/29	0.066
Hypertension (yes/no)	36/4	33/0	0.062
Smoking (yes/no)	16/24	13/20	0.958
H2 receptor blockers (yes/no)	1/39	0/33	0.360
PPI (yes/no)	1/39	0/33	0.360
Phosphate binders (yes/no)	37/3	32/1	0.404
Oral iron supplementation (yes/no)	3/37	3/30	0.805
HD vintage (month)	82.85 (52.85, 127.82)	50.70 (21.95, 68.15)	0.000
Hemoglobin (g/l)	110.22 ± 10.64	109.18 ± 8.59	0.644
Serum creatinine (μmol/l)	863.00 (755.50, 1016.00)	993.00 (817.00, 1381.00)	0.010
Serum calcium (mmol/l)	2.31 ± 0.19	2.26 ± 0.18	0.299
Serum phosphate (mmol/l)	1.72 ± 0.48	1.77 ± 0.57	0.647
Serum albumin (g/l)	40.79 ± 3.02	41.31 ± 2.92	0.461
Serum usCRP (mg/l)	3.41 (1.45, 8.10)	2.55 (1.30, 6.72)	0.451
Serum LDL-C (mmol/l)	1.89 ± 0.51	2.09 ± 0.62	0.150
Serum HDL-C (mmol/l)	0.89 (0.78, 1.08)	0.79 (0.70, 1.01)	0.098
Serum cholesterol (mmol/l)	1.84 (1.10, 2.76)	2.02 (1.12, 3.04)	0.431
Serum triglycerides (mmol/l)	3.57 ± 0.84	3.79 ± 0.82	0.255
Serum ALP (mmol/l)	94.50 (77.25, 116.75)	89.00 (67.50, 117.50)	0.327
Serum iPTH (pg/ml)	321.60 (183.35, 617.47)	224.60 (109.15, 475.95)	0.088
Diets:			
Proteins (g/d)	54.57 ± 17.36	51.64 ± 14.21	0.506
Fats (g/d)	60.45 ± 26.74	55.68 ± 23.39	0.493
Carbohydrates (g/d)	223.71 ± 84.34	203.09 ± 59.88	0.318
Fibers (g/d)	9.11 ± 3.08	9.19 ± 3.78	0.935
AAC scores	8.00 (6.00, 11.75)	1.00 (0.00, 2.00)	0.000

HD: hemodialysis patients; BMI: body mass index; DM: diabetes mellitus; CVD: cardiovascular diseases; H2: histamine; PPI: proton pump inhibitors; usCRP: ultrasensitive C reactive protein; LDL-C: low-density lipoprotein cholesterol; HDL-C: high-density lipoprotein cholesterol; ALP: alkaline phosphatase; iPTH: intact parathyroid hormone; AAC: abdominal aortic calcification.

A total of 3,740,283 reads and 1200 OTUs were obtained from 73 samples. The samples contained a mean of 51,236 reads and 228 OTUs. The samples were randomly flattened according to the minimum number of sample sequences to avoid analysis deviation. A total of 1200 OTUs were obtained, and each sample contained 31,404 reads. A total of 1200 OTUs were shared by the two groups, with 182 and 150 OTUs unique to the high AAC and low AAC score groups, respectively.

### 3.2. Alpha diversity analysis and analysis of microbiota composition

The proportions of bacterial species in the high AAC and low AAC score groups were evaluated by Good’s species coverage index, which revealed that the difference between the two groups was not significant (*p* = 0.070) (Table 2). The coverage index in both groups was higher than 0.99, which confirmed that the test results covered most bacterial species in the fecal sample of HD patients. The Chao1 index showed that the species abundance was significantly lower in the high AAC score group than in the low AAC score group (*p* = 0.044; Table 2). The Shannon index showed that the diversity in the high AAC score group was reduced compared with that in the low AAC score group; however, there was no significant difference between the two groups (*p* = 0.050; Table 2).

**Table 2.** Comparison of *α* diversity in gut microbiota between the high and low AAC score groups in hemodialysis patients.

Items	The high AAC score group（*n* = 40）	The low AAC score group（*n* = 33）	*p* Value
Chao1	263.50 ± 93.73	307.54 ± 80.24	0.044
Shannon	2.92 ± 0.65	3.18 ± 0.54	0.050
Coverage	99.85 × 10^−2^ ± 5.43 × 10^−4^	99.82 × 10^−2^ ± 5.03 × 10^−4^	0.070

AAC: abdominal aortic calcification.

**Figure 2.**
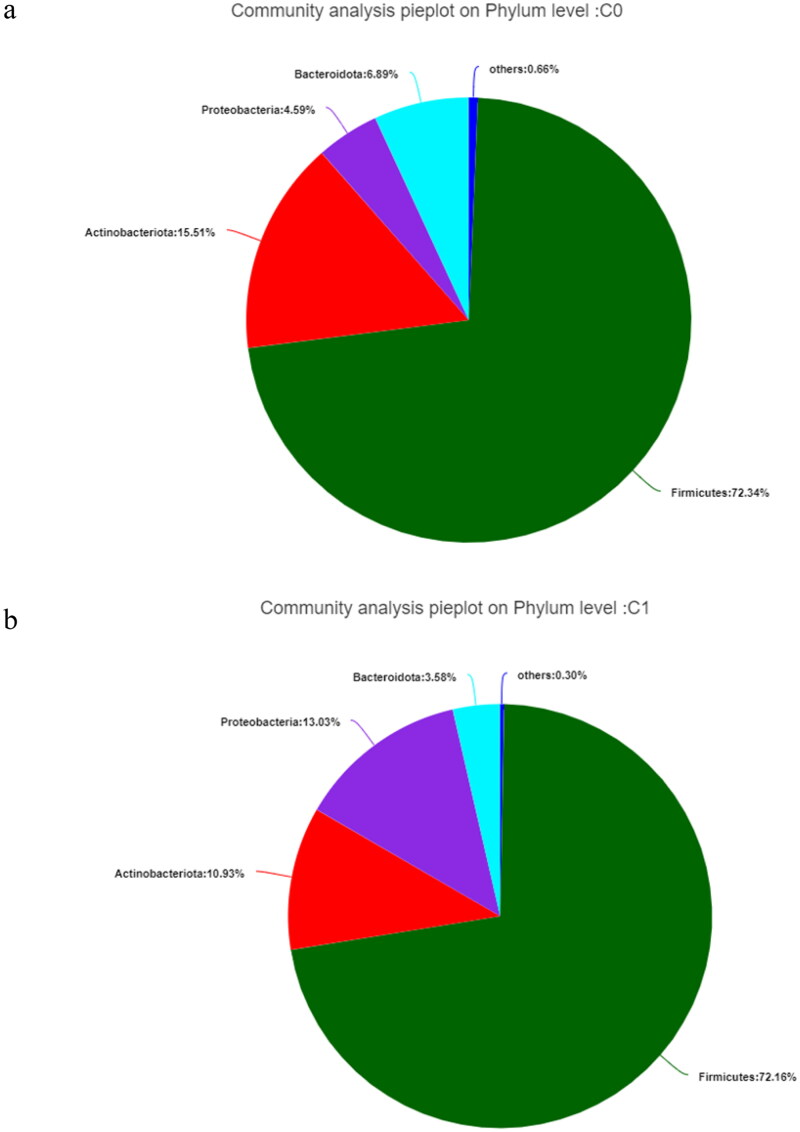
The gut microbiota at the phylum level in hemodialysis patients. C0: the low AAC score group; C1: the high AAC score group; AAC: abdominal aortic calcification. 2a. The gut microbiota at the phylum level in the low AAC score group (C0) in hemodialysis patients. 2b. The gut microbiota at the phylum level in the high AAC score group (C1) in hemodialysis patients.

Analysis of community composition showed that the gut microbiota mainly contained *Firmicutes*, *Actinobacteriota*, *Proteobacteria*, and *Bacteroidota* at the phylum level, regardless of the study group (Figure 2). *Firmicutes* were the most abundant in both groups, followed by *Proteobacteria*, *Actinobacteriota*, and *Bacteroidota* in the high AAC score group (see Figure 2).

### 3.3. Beta diversity analysis and comparison of gut microbiota composition between different VC groups

**Figure 3.**
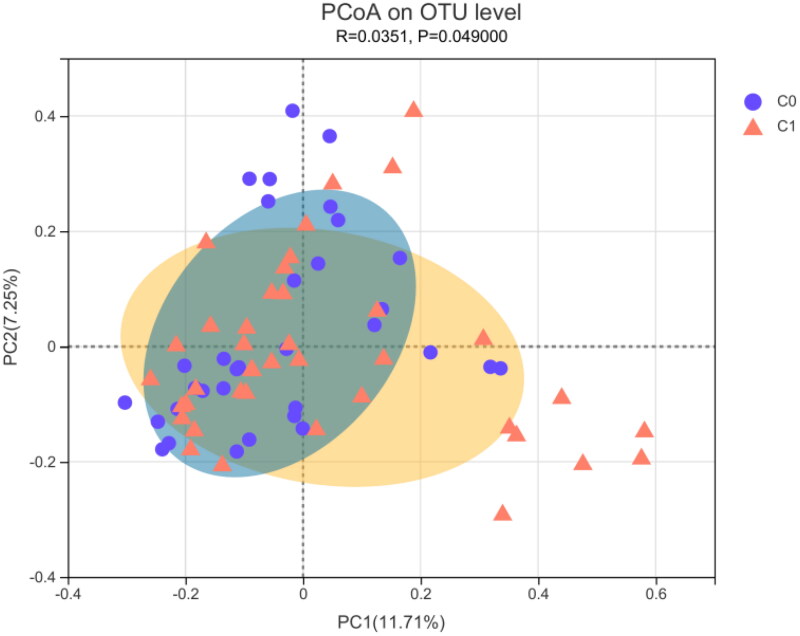
β-diversity measured by PCoA in gut microbiota between the high AAC score group (C1) and the low AAC score group (C0) in hemodialysis patients. C0: the low AAC score group; C1: the high AAC score group; AAC: abdominal aortic calcification.

PCoA analysis of the Bray–Curtis distance was performed to visualize the bacterial composition dissimilarity between the two groups (*R* = 0.0351, *p* = 0.049; Figure 3). Figure 3 shows that the clusters for the two groups were significantly different (PERMANOVA, *p* = 0.033).

**Figure 4.**
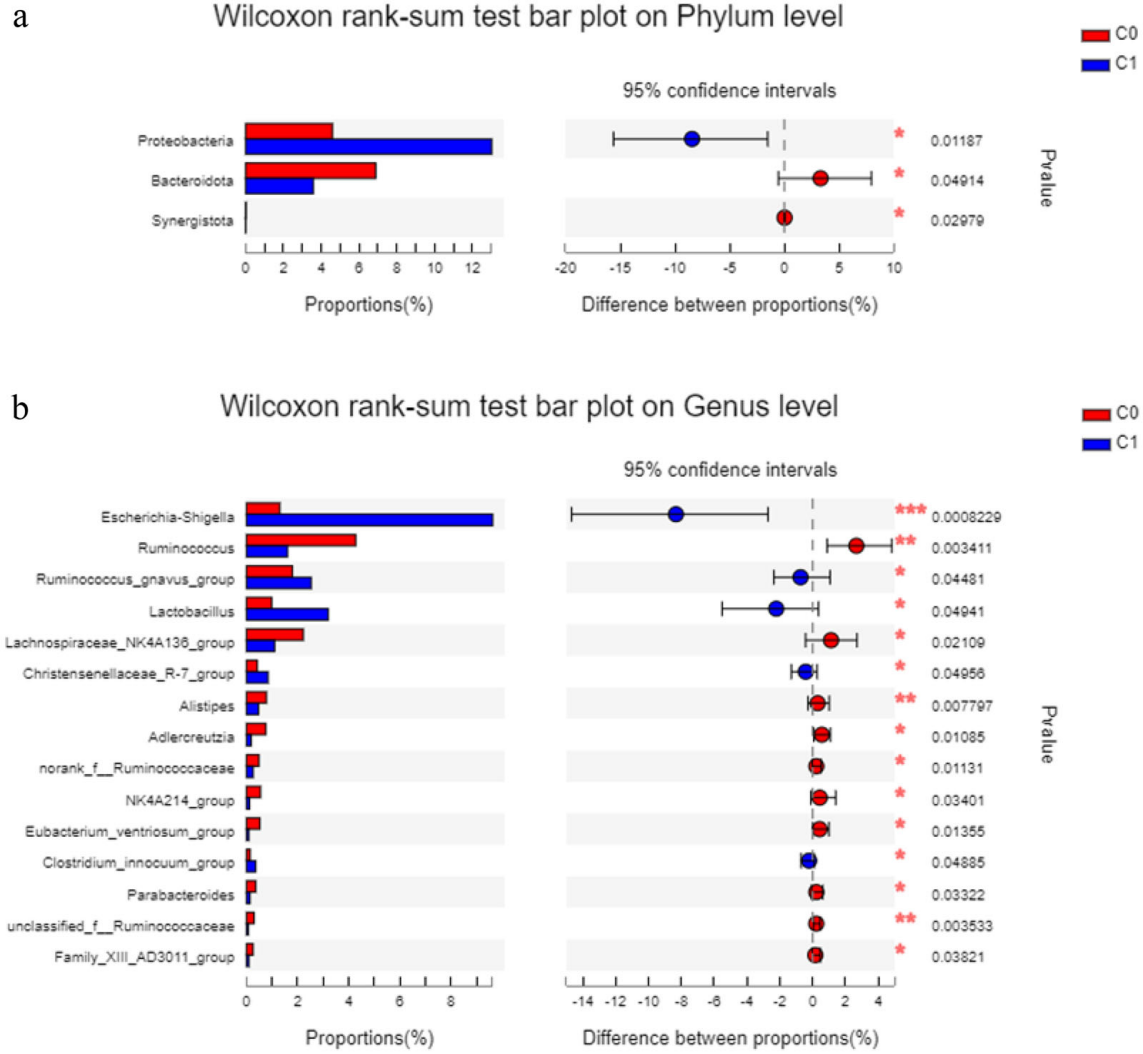
The differences in relative abundance in gut microbiota between the high AAC score group (C1) and the low AAC score group (C0) in hemodialysis patients. C0: the low AAC score group; C1: the high AAC score group; AAC: abdominal aortic calcification. 4a. Phylum-level differences between the high AAC score group (C1) and the low AAC score group (C0) in hemodialysis patients. 4b. Genus-level differences between the high AAC score group (C1) and the low AAC score group (C0) in hemodialysis patients.

As shown in Figure 4, *Proteobacteria* was more abundant in the high AAC score group than in the low AAC score group (*p* = 0.012), and *Bacteroidota* and *Synergistota* were less abundant in the high AAC score group than in the low AAC score group (*p* = 0.049 and *p* = 0.030, respectively) at the phylum level. *Escherichia-Shigella, Ruminococcus gnavus group*, and *Lactobacillus* were more abundant in the high AAC score group than in the low AAC score group at the genus level. *Ruminococcus* and *Lachnospiraceae_NK4A136_group* were significantly enriched in the low AAC group at the genus level.

### 3.4. Correlation analysis between gut microbiota and VC or other clinical markers

**Figure 5.**
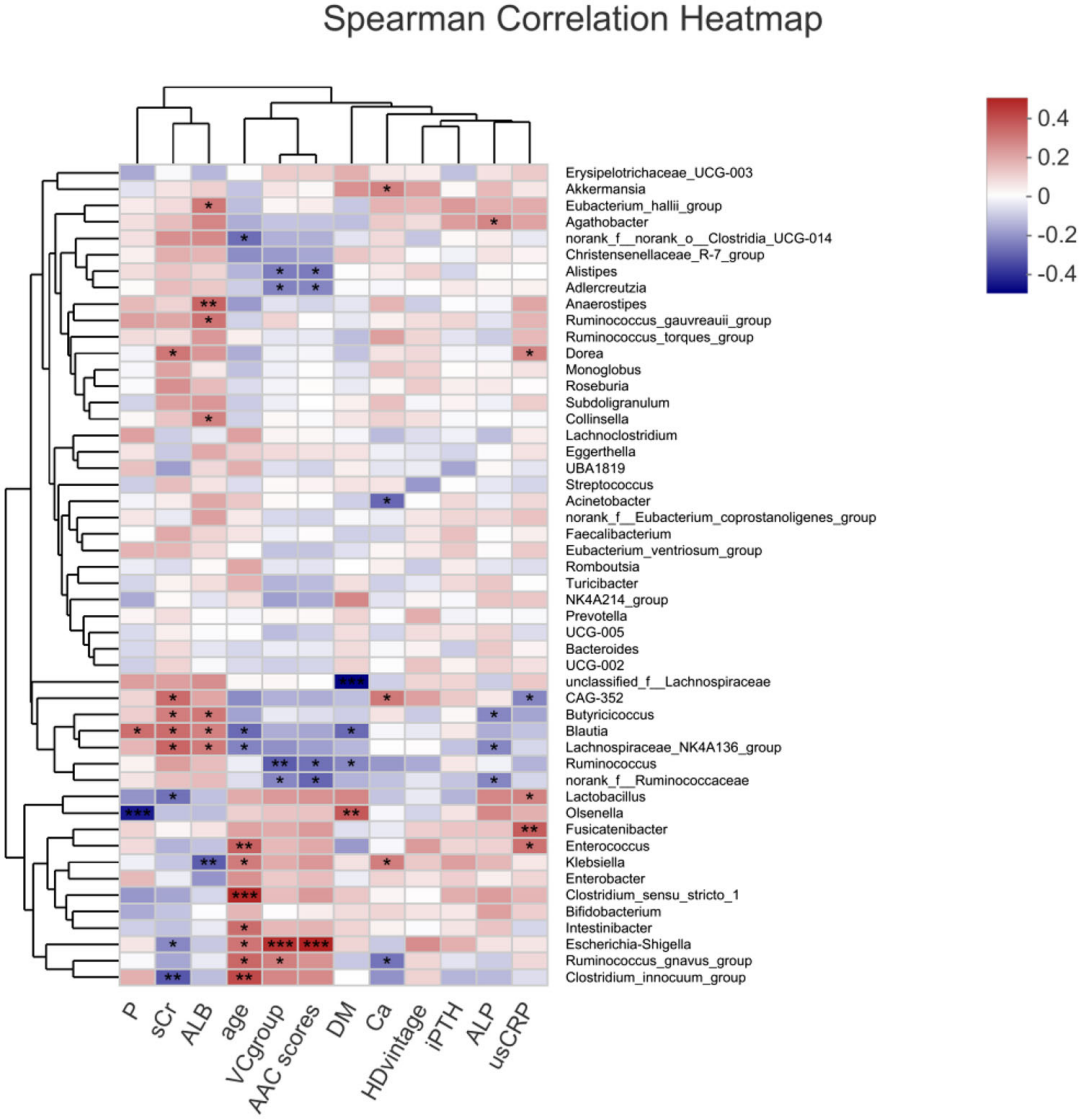
Heatmap showing the correlation between gut microbiota and the VC group or other parameters in hemodialysis patients. P: serum phosphate; sCr: serum creatinine; ALB: serum albumin; VC group: vascular calcification group, the high AAC score group = 1 and the low AAC score group = 0; DM: diabetes mellitus, with DM = 1, without DM = 0; Ca: serum calcium; iPTH: serum intact parathyroid hormone; ALP: serum alkaline phosphatase; usCRP: serum ultrasensitive C reactive protein; AAC: abdominal aortic calcification. Red: positive correlation; Blue: negative correlation, *p < 0.05; **p < 0.01.

**Figure 6.**
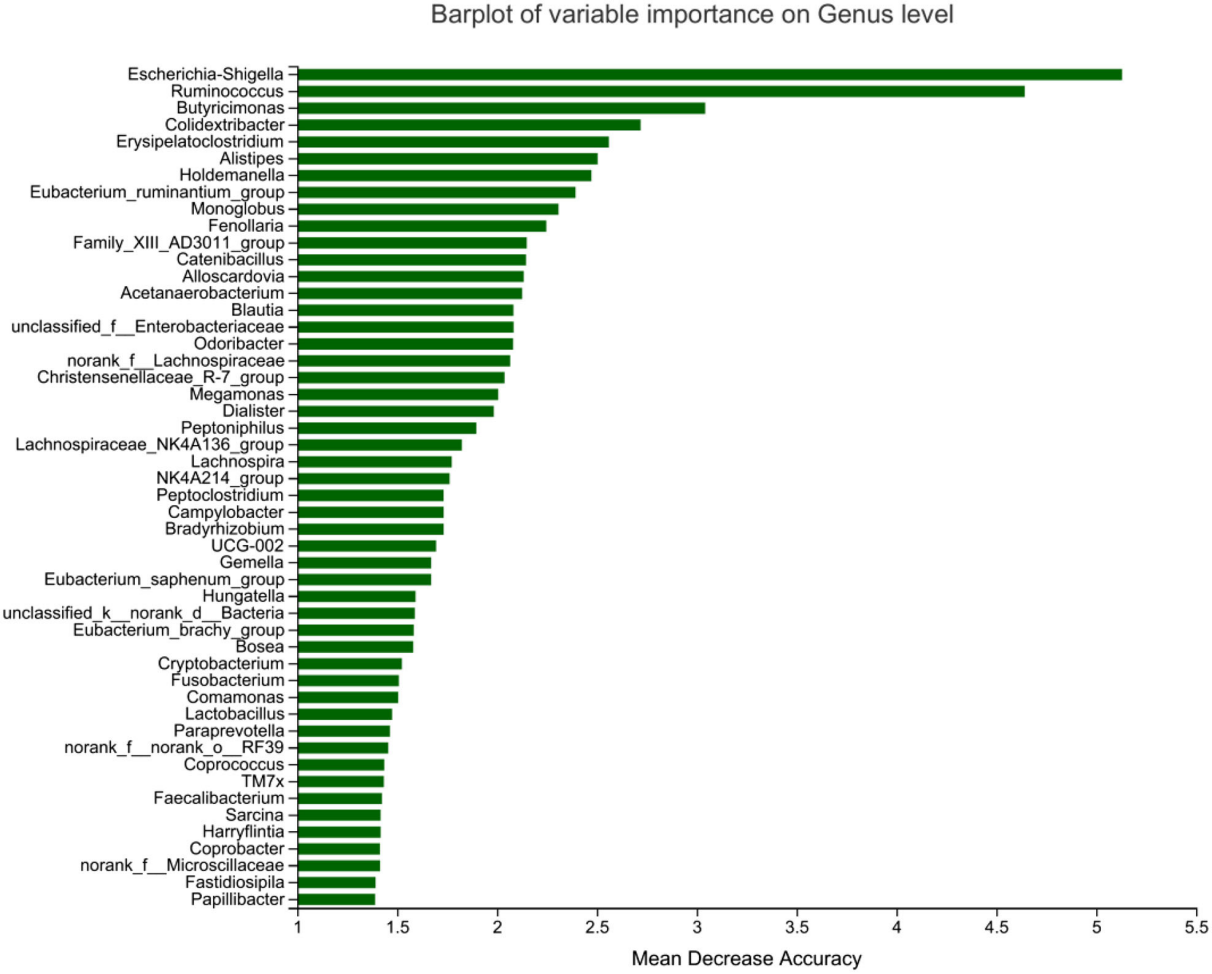
Random forest analysis of the gut microbiota between the high and low AAC score groups in hemodialysis patients. Species importance ranking graph. The Y-axis is the measurement standard of species importance, and the X-axis is the measurement value/standard deviation value of species importance. The Y-axis corresponds to species names sorted by importance. AAC: abdominal aortic calcification.

Heatmap results of the species correlation analysis showed that *Escherichia-Shigella* and *Ruminococcus_gnavus_group* were positively correlated with VC, and *Ruminococcus, Adlercreutzia*, *Alistipes,* and *norank_f__Ruminococcaceae* were negatively correlated with VC (Figure 5). Similar results were obtained for gut microbiota and AAC scores. Random forest analysis uses the decision tree method to evaluate the importance of variables. *Escherichia-Shigella* had the greatest influence on VC in HD patients, followed by *Ruminococcus* and *Butyricimonas* (Figure 6).

The heatmap (Figure 5) also revealed that there was no relationship between any gut microbiota and HD vintage and that no gut bacteria related to serum creatinine, calcium, phosphorus, or iPTH were associated with VC positively or negatively at the same time. In addition, several significant correlations between the relative abundance of gut microbiota and blood biochemical indicators were noted. *Blautia* was positively associated with serum creatinine and albumin and negatively associated with age. *Dorea* was positively associated with serum creatinine. *Enterococcus*, *Lactobacillus*, and *Fusicatenibacter* were positively associated with serum usCRP.

### 3.5. Independent determinant factors of a high AAC score by multiple regression analysis in HD patients

A logistic regression model was performed to identify the independent determinant factors associated with VC in the study. The variables that were significantly related to VC in Table 1 (age, HD duration, and serum creatinine) and the OTU levels of the two main related bacteria (*Escherichia-Shigella* and *Ruminococcus*) entered the analysis as candidate variables. Ultimately, serum creatinine was excluded from the model. Age, HD vintage, *Escherichia-Shigella*, and *Ruminococcus* were independently associated with a high AAC score (see Table 3).

**Table 3.** Independent determinant factors for VC by multiple regression analysis in hemodialysis patients.

Variables	*B*	*p* Value	Exp(B)/OR	95% CI for Exp(B)
HD vintage (months)	0.022	0.010	1.022	1.005–1.039
Age (years)	0.073	0.006	1.075	1.021–1.132
*Escherichia-Shigella*	0.0003	0.081	1.000	1.000–1.001
*Ruminococcus*	−0.001	0.027	0.999	0.997–1.000
Constant	−5.007	0.003	0.007	–

VC: vascular calcification; B: coefficient; OR: odds ratio; CI: confidence interval; HD: hemodialysis.

Age, HD vintage, serum creatinine, *Escherichia-Shigella*, and *Ruminococcus* were initially included in the multivariate logistic regression model to determine the independent influencing factors of VC (using backward conditional).

### 3.6. Predictive functional analysis

**Figure 7.**
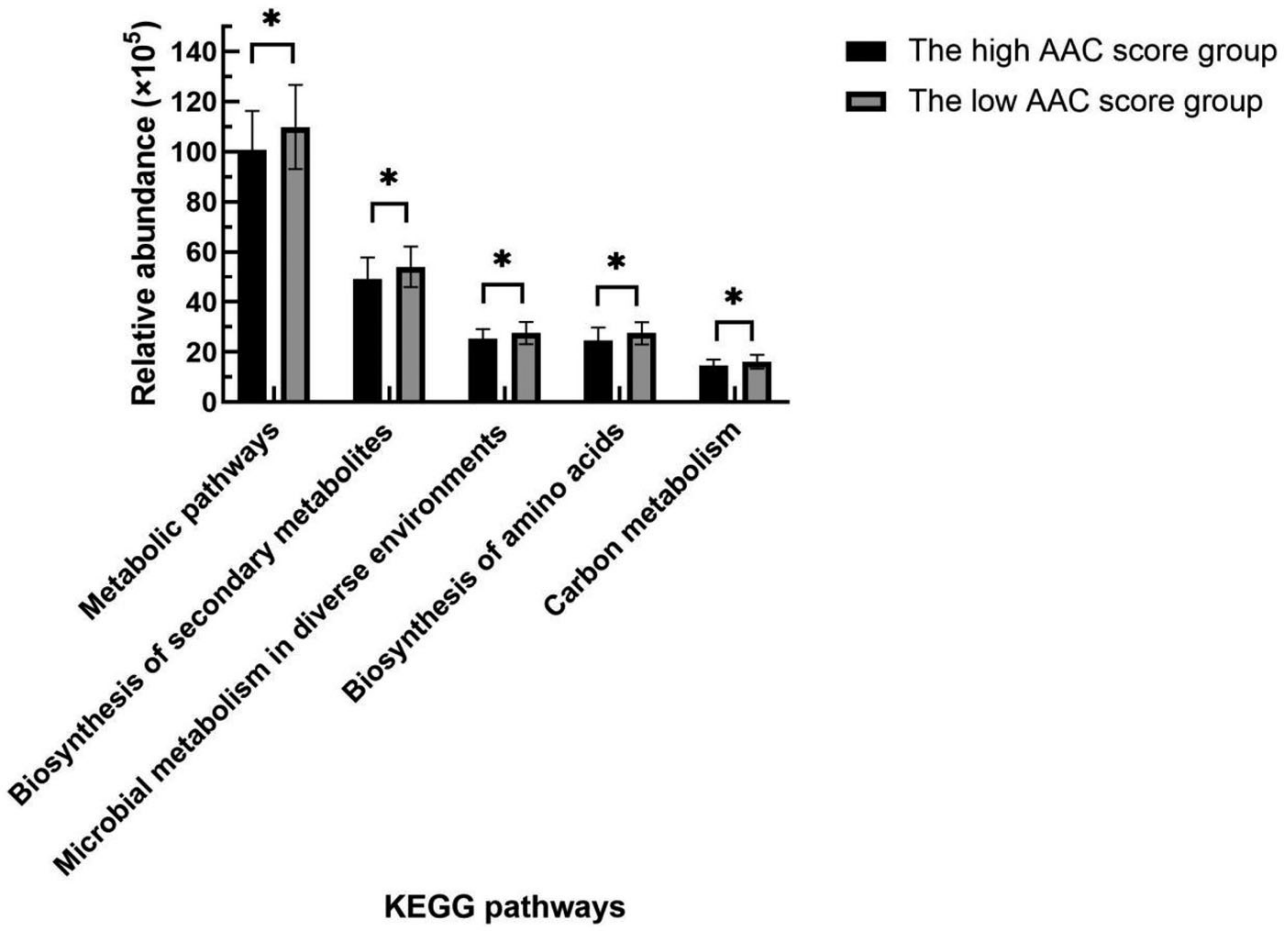
Comparison of functional predictions for the fecal microbiome between the high and low AAC score groups in HD patients by PICRUSt analysis at KEGG level 3 based on 16S sequencing data. AAC: abdominal aortic calcification; HD: hemodialysis; PICRUSt: phylogenetic investigation of communities by reconstruction of unobserved states; KEGG: Kyoto Encyclopedia of Genes and Genomes. *p < 0.05.

PICRUSt was used to predict the functions of the most abundant gene sequences identified in the fecal microbiota based on the constructed OTUs according to the KEGG database. Compared with the patients with low AAC scores, HD patients with high AAC scores mainly showed weakened carbon metabolism, biosynthesis of amino acids, biosynthesis of secondary metabolites, and metabolic pathways (see Figure 7).

## 4. Discussion

In our study, we found that there was a difference in gut microbiota between the different VC groups in HD patients. *Escherichia-Shigella* was positively correlated with VC and had the greatest influence on VC independently, and *Ruminococcus* was negatively correlated with VC independently and had the second greatest influence on VC in HD patients.

Our results showed that the gut microbiota in the high AAC score group was different from that in the low AAC score group in HD patients. Based on the alpha diversity analysis, the abundance of gut microbiota in the high AAC score group was significantly lower than that in the low AAC score group. The diversity of gut microbiota in the high AAC score group was lower, but the difference was not significant. However, Ana Merino-Ribas et al. found similar values of alpha diversity in gut microbial communities when comparing PD patients with and without VC [17]. This finding was not the same as our results in HD patients, which may be related to different dialysis modalities. To the best of our knowledge, no other studies on gut microbiota and VC in ESRD patients have been reported until now. Other existing studies only suggest that the gut microbiota in HD or PD patients was different from that in healthy controls, and most showed that the abundance and diversity of gut microbiota were lower in the HD group than in controls [12,18]. Takumi Toya et al. found that the abundance and diversity of gut microbiota were lower in patients with coronary artery disease (CAD) than in those without CAD [19]. It seems that the abundance and diversity of gut microbiota decrease in most disease states.

We observed that there were differences in the species composition and abundance between the high and low AAC score groups in HD patients based on beta diversity analysis. However, *Firmicutes*, *Actinobacteriota*, *Proteobacteria*, and *Bacteroidota* were still the dominant bacteria in all groups at the phylum level. This is consistent with Ana Merino-Ribas’ observations in PD patients [17] and some other studies of ESRD patients [13,18]. In our study, compared with patients with low AAC scores, patients with high AAC scores showed an increased abundance of *Proteobacteria* and decreased abundances of *Bacteroidota* and *Synergistota* at the phylum level and increased abundances of *Escherichia-Shigella, Ruminococcus_gnavus_group*, and *Lactobacillus* and decreased abundances of *Ruminococcus* and *Lachnospiraceae_NK4A136_group* at the genus level. However, in PD patients [17], Ana Merino-Ribas did not find gut microbiota differences at the phylum level, and only *Coprobacter*, *Coprococcus 3*, *Lactobacillus*, and *Eubacterium eligens* were increased in the VC group. These findings are different from our results and may be due to different dialysis modalities, races, dietary habits, and lifestyles.

*Proteobacteria* are a minor constituent within a balanced gut-associated microbial community. The abundance of *Proteobacteria* was increased in HD patients compared with healthy controls [13. Dysbiotic expansion of *Proteobacteria* is a microbial signature of epithelial dysfunction [20]. *Escherichia-Shigella,* belonging to the phylum *Proteobacteria,* can be pathogenic and are often labeled pathobionts [21]. A previous study in HD patients found that *Escherichia* was substantially more abundant in the protein energy wasting (PEW) group than in the non-PEW group [22]. In our study, we showed that *Escherichia-Shigella* was positively correlated with VC and had the greatest influence on VC in HD patients. Flannigan KL et al. demonstrated that the expansion of *Proteobacteria* (specifically *Escherichia-Shigella*) was accompanied by gene enrichment for multiple bacterial enzymes involved in the biosynthesis of lipopolysaccharide (LPS) and increased levels of LPS in the feces and serum in mice [21]. LPS, also called endotoxin, is a key factor in the initiation and progression of atherosclerosis through mediation of endothelial cell injury, promotion of recruitment of monocytes, transformation of macrophages to foam cells, and procoagulant activity. Previous studies have shown that elevated endotoxin levels are a strong risk factor for the development of atherosclerosis [23]. LPS may promote VC through interaction with monocyte/macrophages releasing inflammatory cytokines in CKD rats, and low LPS/toll-like receptor 4 (TLR4) signaling may directly promote VC under chronic stimulation *in vivo* [24]. In addition, *Proteobacteria* can break down protein/amino acids in food [25]. Uremic toxins, such as IS and PCS are produced by the metabolism of amino acids, such as tryptophan and tyrosine, respectively, involving gut *Escherichia-Shigella*. Previously, Barreto et al. disclosed a positive association between IS, PCS, and VC in CKD patients [26,27] Recently, Britt Opdebeeck et al. found that long-term exposure to IS and PCS significantly increased VC in CKD rats *via* activation of inflammation and coagulation pathways [7]. Thus, it can be seen that the increase in *Escherichia-Shigella* is related to VC, which may be accelerated by the increases in LPS production and inflammation and by the increases in IS and PCS, the metabolites from the amino acid catabolism associated with the bacteria.

We found that *Ruminococcus* was negatively correlated with VC and played the second-most important role in VC in HD patients. However, there was no bacteria with a significantly negative relationship to VC in Ana Merino-Ribas’ study of PD patients [17]. *Ruminococcus* are gram-positive anaerobic bacteria belonging to the family *Ruminococcaceae* and the phylum *Firmicutes,* which are widely recognized for their capacity to hydrolyze and ferment many structural and storage polysaccharides to produce short-chain fatty acids (SCFAs), such as butyrate [28,29]. Recent studies found that SCFAs seem to contribute to the improvement of vascular phenotypes [1,30]. SCFAs may have immunomodulatory effects. Inflammatory cells, such as neutrophils, macrophages, dendritic cells, and T cells are responsive to SCFA treatment, which is consistent with the anti-inflammatory role of SCFAs in a wide range of inflammatory diseases and the reduction of VC [30,31].

In addition to *Escherichia-Shigella*, which was positively correlated with VC and had the greatest impact on VC in our study, several other bacteria were positively related to VC. In this study, we found that *Ruminococcus gnavus group* had increased abundance in high AAC scores and was positively correlated with VC. *Ruminococcus gnavus group,* which is associated with Crohn’s disease and CAD [19], produces an inflammatory polysaccharide [32]. It belongs to the family *Lachnospiraceae* and is different from the genera *Ruminococcus* and *norank_f_Ruminococcaceae,* which both belong to the family Ruminococcaceae. The inflammatory polysaccharide produced by the *Ruminococcus gnavus group* induces the production of inflammatory cytokines, such as tumor necrosis factor alpha (TNF-α) by dendritic cells [32]. TNF-α was inversely associated with estimated glomerular filtration rate (eGFR), indicating a role for inflammation in CKD development and progression. Inflammatory cytokines can also promote VC [33]. TNF-α is also an inhibitor of bone formation, tilting the balance toward bone resorption with subsequent bone loss [34]. In our study, we also found an increased abundance of *Lactobacillus* in the high AAC score group, which is consistent with a study in PD patients [17]. *Lactobacillus* plays an important role in maintaining the integrity of the intestinal barrier and regulating the immune system. However, certain strains can also turn into pathobionts in the environment of a predisposed host. Previous reports showed that certain strains of *Lactobacillus* can break through a dysfunctional gut barrier, colonize internal tissues, such as the spleen or liver and promote inflammatory responses in host tissues [35]. HD patients have immune system disorders; therefore, we speculate that *Lactobacillus* promotes the inflammatory response in HD patients, leading to VC.

In addition to *Ruminococcus*, which was negatively correlated with VC and played the second-most important role in VC in our study, several other bacteria were negatively associated with VC. We found that *Adlercreutzia* and *Alistipes* were negatively correlated with VC. Yu et al. found that the increased abundance of *Adlercreutzia* may be associated with the increased production of SCFAs in the intestine [36]. *Alistipes*, a relatively new genus of bacteria belonging to the phylum *Bacteroidetes*, may have protective effects against some diseases, such as liver fibrosis, colitis, cancer immunotherapy, and CVD. It is also an SCFA-producing bacterium [37]. *Bacteroidetes* are involved in the biotransformation of sugars, bile acids, and steroids. Therefore, it is possible that a decrease in *Bacteroidetes* can lead to a decrease in SCFA production, which promotes VC and affects cardiovascular outcomes [37,38]. Luo et al. also found that *Bacteroidetes* were significantly deceased in HD patients and that *Bacteroides* was associated with cardiovascular mortality [10]. Another bacterium, *Lachnospiraceae_NK4A136_group,* is generally considered an SCFA producer, and its abundance is negatively correlated with inflammation. Increased *Lachnospiraceae_NK4A136_group* abundance and fecal SCFA contents were linked to decreases in TLR4 signaling and LPS levels [39]. This is consistent with our results that *Lachnospiraceae_NK4A136_group* had decreased abundance in the high AAC score group. *Butyricimonas* was first found in rat feces and is also an SCFA-producing microbe [40]. We found that *Butyricimonas* had the third greatest effect on VC in HD patients in our study.

In addition, the PICRUSt analysis at KEGG level 3 based on 16S sequencing data showed that the biosynthesis of amino acids, biosynthesis of secondary metabolites, carbon metabolism, etc., in the high AAC score group decreased significantly, indicating that most of the anabolism of amino acids, which was related to the gut microbiota affecting VC, was weakened. Subhashree Shivani’s study found that in a healthy cohort, many biosynthesis pathways were highly enriched, whereas in a dialysis cohort, many degradation or catabolism pathways were highly functional [41]. However, the metabolic pathways related to the gut microbiota should be further confirmed more precisely by metagenomics.

According to our results, *Escherichia-Shigella* and some special bacteria involved in protein metabolism increased significantly in the high AAC score group, which may aggravate VC by producing LPS, increasing the inflammatory response, or increasing the levels of uremic toxins such as IS and PCS. However, *Ruminococcus*, *Bacteroidetes* and some other bacteria involved in the metabolism of sugars and bile acids were significantly reduced, which may weaken the protective mechanism of VC through abnormal metabolism in SCFA or bile acid. Therefore, the underlying mechanisms merit further investigation. It is suggested that reducing the abundance of *Escherichia-Shigella* and increasing the abundance of *Ruminococcus* and *Bacteroidetes*, blocking the inflammatory mechanism and increasing SCFA may become therapeutic targets to improve VC in dialysis patients.

Moreover, in the multivariate regression analysis, *Escherichia-Shigella* and *Ruminococcus* were independent related factors of VC. We did not observe the same microbiota related to serum calcium, phosphorus, or iPTH. This revealed that the gut microbiota likely does not affect VC by directly influencing the metabolism of calcium-phosphorus. In our study, *Blautia* and *Dorea* were associated with serum creatinine or albumin, which was consistent with Luo’s study [10]. This result suggested that the gut microbiota and nutritional state interact with each other in HD patients [22]. The bacterial taxa related to age and usCRP were different from some studies [22,41], which may be due to the different populations evaluated. Further studies are needed to clarify this hypothesis.

In this study, we found that age and HD vintage were significantly different between the high and low AAC score groups but did not find differences in serum calcium, serum phosphate, or serum iPTH between the two groups. This may be because we only used a single level of calcium, phosphorus, and iPTH, whereas VC is a pathological process in long-term excessive calcium, phosphate, and iPTH loading. In addition, our patients’ well-controlled serum calcium, phosphorus, and iPTH levels and relatively small sample sizes may limit our ability to identify correlations.

Altogether, we found that there was a difference in gut microbiota between the different VC groups in HD patients. However, there were several limitations. First, this was a cross-sectional observational study, and it was difficult to derive causal associations. Second, our study was conducted at a single center, and the sample size of this study was relatively small; therefore, selection bias cannot be avoided completely. Larger sample sizes and a multicenter research design will be required in further studies. Third, the dietary assessments were relatively crude in our study and should be made more precise in the future to rule out confounding factors. Fourth, fecal samples from patients were not collected multiple times, which could better observe the dynamic changes in gut microbiota. Fifth, in analyzing the difference in relative abundance of gut microbiota between different VC groups, the Wilcoxon rank-sum test was not corrected for multiple comparisons. Therefore, the results from this might be by chance due to multiple inferences made. However, this limitation may be balanced by the following heatmap correlation analysis, random forest analysis and multiple regression analysis, which identified the same significant bacteria related to VC.

## 5. Conclusion

Our study provided clinical evidence that there was a difference in gut microbiota between the different VC groups in HD patients. *Escherichia-Shigella,* an LPS-producing bacterium, was positively correlated with VC and had the greatest influence on VC. *Ruminococcus*, an SCFA-producing bacterium, was negatively correlated with VC and had the second-greatest influence on VC in HD patients. The underlying mechanism of gut microbiota and VC is worth further exploration. Our study results may hint at new target therapeutic pathways for VC in the future.

## Data Availability

All data generated or analyzed during this study are included in this article. Further inquiries can be directed to the corresponding author.
